# Peripheral PDLIM5 expression in bipolar disorder and the effect of olanzapine administration

**DOI:** 10.1186/1471-2350-13-91

**Published:** 2012-10-02

**Authors:** Mohd Aizat Zain, Suffee Nusrat Jahan, Gavin P Reynolds, Nor Zuraida Zainal, Sharmilla Kanagasundram, Zahurin Mohamed

**Affiliations:** 1 The Pharmacogenomics Laboratory, Department of Pharmacology, University of Malaya, 50603, Kuala Lumpur, Malaysia; 2 Department of Psychological Medicine, University of Malaya, 50603, Kuala Lumpur, Malaysia; 3 Biomedical Research Centre, Sheffield Hallam University, City Campus, Howard Street, Sheffield, S11WB, UK; 4 Department of Pharmacology, Department of Psychology Medicine, University of Malaya, 50603, Kuala Lumpur, Malaysia

**Keywords:** Bipolar disorder, Manic, Leukocytes, Olanzapine

## Abstract

**Background:**

One of the genes suggested to play an important role in the pathophysiology of bipolar disorder (BPD) is *PDLIM5*, which encodes LIM domain protein. Our main objective was to examine the effect of olanzapine treatment on *PDLIM5* mRNA expression in the peripheral blood leukocytes of BPD patients.

**Methods:**

We measured the expression of *PDLIM5* mRNA from 16 patients with BPD Type I after 0, 4, and 8 weeks of treatment with olanzapine using quantitative real-time PCR. The Young Mania Rating Scale was used to evaluate the severity of manic symptoms in BPD patients. We also compared *PDLIM5* mRNA expression in treatment-naïve BPD patients with that in healthy control subjects.

**Results:**

No significant difference was found in *PDLIM5* mRNA expression between patients before olanzapine treatment and following 4 and 8 weeks of treatment (*p*>0.05). Although we observed a significant reduction in the severity of manic symptoms in all BPD patients (*p*<0.05), the effectiveness of the medication did not significantly correlate with the expression of *PDLIM5* mRNA (*p*>0.05). Interestingly, *PDLIM5* mRNA expression differed significantly between treatment-naïve BPD patients and healthy control subjects (*p*=0.002).

**Conclusion:**

*PDLIM5* mRNA expression did not appear to be a reflection of the efficacy of olanzapine in reducing the manic symptoms of BPD. The significant difference in expression of *PDLIM5* mRNA in the peripheral blood leukocytes of treatment-naïve BPD patients versus that of healthy control subjects, however, suggests that it may be a good biological marker for BPD.

## Background

Bipolar disorder (BPD) is a mental illness that specifically affects mood and has an estimated prevalence of 0.8-2.6% [1]. Strong evidence from twin, adoption, family, and linkage studies has shown that BPD is associated with genetic factors, with an estimated heredity of 63%[1]. On the basis of evidence reported in genetic association and expression studies, *PDLIM5* is a promising candidate gene for BPD [2].

The *PDLIM5* gene lies on chromosome 4q22 and consists of a PDZ (post-synaptic density-95/discs large/zone occludens-1) domain and three LIM (Lin-11 Isl-1 mec03) domains. LIM domains are cysteine-rich double zinc fingers composed of 50 to 60 amino acids that are involved in protein-protein interactions such as cytoskeleton organization, cell lineage specification, organ development, and oncogenesis. LIM domains are also members of the Enigma class of proteins, including the Enigma homologue (ENH), a family of proteins that possess a 100-amino acid PDZ domain in the N terminus and one to three LIM domains in the C terminus. The *PDLIM5* gene is ubiquitously expressed in the brain and its cellular localization is identical to that of synapsin I, which is known to be involved in neurotransmitter release. The LIM domain is an intermediate protein that can interact with various isoforms of protein kinase C (PKC). LIM domains have been reported to modulate intracellular calcium levels by linking N-type calcium channels with PKC epsilon, thereby promoting the phosphorylation-dependent modulation of channel properties [3]. The formation of the PKCε-ENH-N-type Ca^2+^ channel complex has been hypothesized to have an important role in the molecular basis and efficiency of cellular signalling [4,5].

In genetic association studies, single nucleotide polymorphisms (SNPs) that are located in the upstream region of *PDLIM5* have been found to be associated with BPD [6,7], schizophrenia [8], and major depressive disorder [9]. In addition, previous studies showed a significant association between BPD and haplotypes of *PDLIM5* polymorphisms located in the upstream region [7,10]. However, other studies failed to replicate these findings, possibly because of genetic heterogeneity or methodological inconsistencies [11-13]. Expression studies had found that the mRNA expression of the *PDLIM5* gene was increased in post-mortem brain tissue [14] and decreased in lymphoblastoid cell lines [14,15] derived from peripheral blood leukocytes (PBLs) of BPD patients. The difference might be caused by a state-related rather than a trait-related change in *PDLIM5* gene action [12].

Alterations in PKC activity have been suggested to play an important role in BPD and may be the core pathological characteristic [16,17]. Novel findings confirmed the molecular function of the LIM domain in regulating PKC activity in a PKC isoform-specific manner [18]. PDLIM5 was also shown to promote shrinkage of dendritic spines that may be associated with cognitive impairment [19]. Previous pharmacotherapy studies showed an interaction between lithium and the LIM domain in improving manic symptoms in BPD through the normalization of PKC activity [16,17]. On the other hand, one study showed no interaction between lithium prophylaxis and the LIM domain [20]. Currently, atypical antipsychotics that have mood stabilizer properties are frequently used to treat BPD because of their improved tolerability and efficacy [21]. Olanzapine, an atypical antipsychotic used in the treatment of BPD, has been shown in several studies to improve the symptoms of mania [22-25]. We hypothesize that this effect of olanzapine reflects in part its indirect interaction with the LIM domain in the signalling cascade, leading to an improvement in manic symptoms by normalizing PKC activity. In this study, we compared *PDLIM5* mRNA expression in BPD patients before olanzapine treatment and at 4 and 8 weeks after treatment. We also compared *PDLIM5* mRNA expression between treatment-naïve BPD patients and control subjects. In addition, we examined the association between *PDLIM5* mRNA expression in treatment-naïve patients with BPD Type I and four SNPs of the *PDLIM5* gene located in the upstream region: rs10008257, rs2433320, rs2433322, and rs2438146.

## Methods

The subjects in this study consisted of 16 patients with BPD Type I (6 males, 10 females; mean age 40.25±14.6) and 16 control subjects (8 females, 8 males; mean age 44.5±10.25). Patients were recruited from the inpatient psychiatric clinic of University of Malaya Medical Centre (UMMC), and control subjects were recruited from the medical clinic of the UMMC. The protocol used in this study was approved by the Medical Ethics Committee of UMMC, and all subjects signed consent forms before they participated in the study. All subjects reported an absence of inter-ethnic marriage for at least three prior generations. Demographic data were recorded, including age, gender, marital status, education, occupation, ethnicity, duration of illness, previous non-compliance, previous substance abuse, and family history of mental illness. Only patients diagnosed with BPD Type I were included in this study. Patient diagnosis was undertaken by a qualified psychiatrist using the Mini International Neuropsychiatric Interview assessment scale [26]. The patients were confirmed as being treatment-naïve or as having no history of psychotropic medication for at least 3 months. The progress of all patients was extensively monitored during the 8 weeks of treatment with olanzapine. Patients were given 5 to 20 mg of oral olanzapine daily, as assessed by the psychiatrist during the 8 weeks of treatment. Severity of illness was evaluated by the psychiatrist using the Young Mania Rating Scale (YMRS) at three time points, namely, before medication and after 4 and 8 weeks of olanzapine treatment. Control subjects were confirmed as having no history of mental illness and no first-degree relatives with mental illness, having no chronic disease, and not taking psychotropic medication or any other medications for chronic diseases.

Patient blood samples were collected in two tubes: a Tempus Blood^TM^ RNA Tube and an ethylenediaminetetraaceticacid (EDTA) tube. The Tempus Blood^TM^ RNA Tube contains 6 ml of Applied Biosystems Stabilizing Reagent, which immediately lyses blood cells after the blood is drawn into the tube, whereas the sample in the EDTA tube was used for DNA extraction. The RNA and DNA were extracted from the blood using the Tempus Blood^TM^ RNA Extraction kit and QIAamp DNA Mini Kit (Qiagen, Hilden, Germany), respectively. All procedures of blood collection, storage, handling, and DNA and RNA extraction of the samples followed standard protocols provided by the manufacturers. The quantity and purity of the RNA and DNA were measured using NanoDrop (NanoDrop Technologies, Wilmington, DE, USA).

Expression of the *PDLIM5* gene was quantified with the StepOnePlus^TM^Real-Time PCR System with the TaqMan® Gene Expression Assay and TaqMan® Gene Expression Master Mix according to the manufacturer’s instructions. Primers and probes were purchased from Applied Biosystems (Assay ID: PDLIM5, Hs00179051_m1). Glyceraldehyde-3-phosphate dehydrogenase was used as an internal control for normalization. Samples were run in real-time PCR in triplicate. We used the comparative C_t_ method to calculate relative changes in *PDLIM5* gene expression following the subject’s response to olanzapine medication. Data were collected and analysed using StepOnePlus^TM^ software version 2.1 (Applied Biosystems, Foster City,CA, USA).

In our preliminary study to investigate the association between genotype and expression level of the *PDLIM5* gene, we genotyped four SNPs of *PDLIM5* (rs10008257, rs2433320, rs2433322, and rs2438146) for the 16 treatment-naïve BPD patients using an inventoried TaqMan probes assay, TaqMan Genotyping Master Mix, and StepOnePlus^TM^Real-Time PCR, following the protocol provided by the manufacturer (Applied Biosystems).

Statistical calculations in this study were performed using SPSS statistical software version 16.0. The distributions of YMRS scores and *PDLIM5* mRNA expression were checked for normality using the Kolmogorov-Smirnov test. The differences between *PDLIM5* mRNA expression in controls and patients, as well as in patients before and after 4 and 8 weeks of olanzapine treatment, were calculated using the Wilcoxon signed rank test. The differences between the YMRS scores, however, were determined using the Wilcoxon signed rank test only in patients before and after 4 and 8 weeks of olanzapine treatment. A Spearman correlation coefficient test was performed to investigate the association between *PDLIM5* mRNA expression and YMRS scores following 8 weeks of treatment. The SHEsis web-based platform was used to calculate linkage disequilibrium between the four SNPs of *PDLIM5*[27]. A one-way analysis of variance test was used to determine the association between *PDLIM5* mRNA expression and genotypes of the four SNPs of the *PDLIM5* gene, as well as between *PDLIM5* mRNA expression and patient demographic data, including duration of illness, previous non-compliance, previous substance abuse, and family history with mental illness, in treatment-naïve BPD patients. Hardy-Weinberg equilibrium was checked for all four SNPs using the Hardy-Weinberg calculator online tools [28].

## Results

The demographic data of the patients in this study are shown in Table 1. Among the 16 BPD patients recruited, 4 were Malay, 8 Chinese, and 4 Indian, whereas among the control subjects, 9 were Malay, 5 Chinese, and 2 Indian. In our preliminary association study, we detected three SNPs of the *PDLIM5* gene in perfect linkage disequilibrium (D’=1.00): rs2433320, rs2438146, and rs2433322. Thus, we analysed only the rs2433320 polymorphism as representative of the other two SNPs. We observed no significant difference between the SNPs of *PDLIM5* and its mRNA expression in treatment-naïve BPD patients (Table 2). As the sample size was small, we did not stratify the samples into the different ethnicities. The genotype distributions for the four SNPs were in Hardy-Weinberg equilibrium for all patients.

**Table 1:** **Demographic data of the16 BPD patients inthis gene expression study**

**Characteristic**		**Frequency**	**Percentage**
Gender	Male	6	33.3
	Female	10	55.6
Marital status	Single	9	50
	Married	5	27.8
	Divorced	2	11.1
Education	Primary	2	11.1
	Secondary	7	38.9
	Tertiary	7	38.9
Occupation	Employed	7	38.9
	Unemployed	9	50
Race	Malay	4	22.2
	Chinese	8	44.4
	Indian	4	22.2
Duration of illness	<5 y	6	33.3
	6-10 y	4	22.2
	11-20 y	2	11.1
	>20 y	4	22.2
Previous non-compliance	Yes	13	72.2
	No	3	16.7
Previous substance abuse	Yes	4	22.2
	No	12	66.7
Family mental illness	Yes	5	27.8
	No	11	61.1

*BPD* bipolar disorder.

**Table 2:** Association between genotypes of *PDLIM5 *gene polymorphisms and *PDLIM5 *mRNA expression in treatment-naïve BPD patients*

**SNP**	**Genotype**	***N***	**Mean**	**SD**	***F***	***p*value**
rs10008257	AA	2	0.3	0.078	0.348	0.713
	AG	5	0.39	0.351		
	GG	8	0.27	0.205		
rs2433320	GG	10	0.34	0.257	2.019	0.176
	GA	4	0.16	0.107		
	AA	1	0.65	-		

*Prior to medication with olanzapine.

*BPD* bipolar disorder, *SNP* single nucleotide polymorphism.

**Figure 1:**
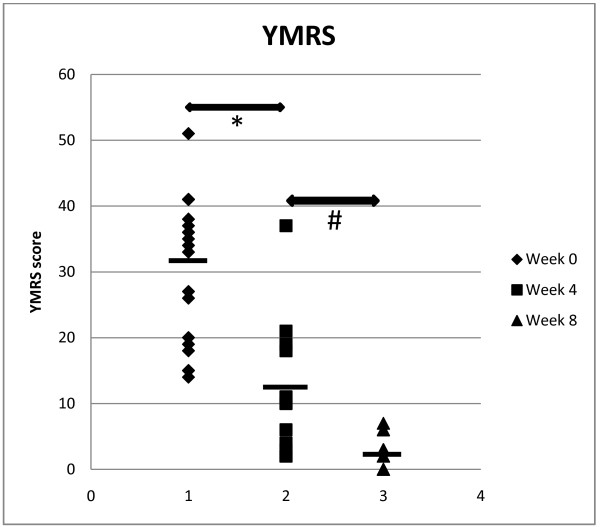
**The mean Young ManiaRating Scale (YMRS) scorebefore (0 weeks) andafter 4 and 8weeks of olanzapine treatmentin patients with bipolardisorder (BPD).** The mean YMRS score was significantly lower after 4 weeks of treatment with olanzapine (mean YMRS score before medication: 30.31±10.81; after 4 weeks of medication: 10.53±9.82; Wilcoxon rank sum test: *p= 0.001). The mean YMRS score of the BPD patients was further significantly reduced after 8 weeks after medication compared with the score after 4 weeks of medication (YMRS score after 8 weeks of medication: 2.5±2.20; Wilcoxon rank sum test: ^#^*p*= 0.005). The YMRS mean scores before and after treatment are indicated by horizontal lines.

**Figure 2:**
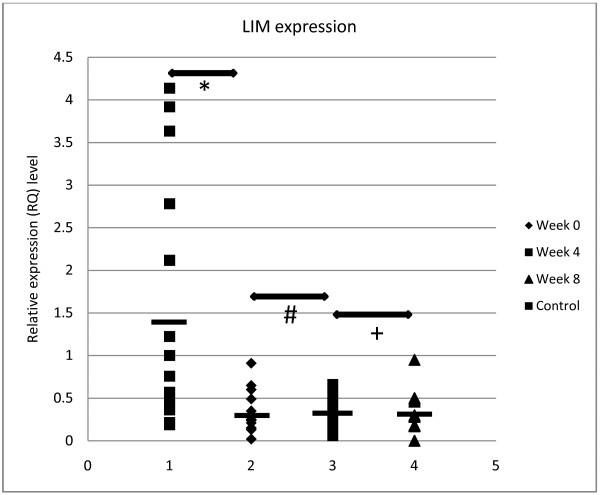
***PDLIM5*mRNA expression level incontrol subjects and patientsat 0, 4, and8 weeks after olanzapinetreatment.** The mean *PDLIM5* mRNA expression level was significantly lower in naïve patients than in control subjects (naïve patients: 0.31±0.245; controls: 1.42±1.42; Wilcoxon rank sum test: *p=0.002). After 4 weeks of treatment, no significant changes were detected in *PDLIM5* mRNA expression when compared with the mean mRNA level in treatment-naïve patients (after 4 weeks of treatment: 0.36±0.195; Wilcoxon rank sum test: *p*=0.826). The *PDLIM5* mRNA expression level after 8 weeks was 0.36±0.244, with no statistical difference when compared with the mRNA level after 4 weeks of treatment (Wilcoxon rank sum test: *p*=0.937). The mean *PDLIM5* mRNA expression level in controls and patients before and after treatment is indicated by horizontal lines.

Both the YMRS scores and *PDLIM5* mRNA expression were in normal distribution (*p*>0.05). We compared the mean YMRS scores of patients before and after taking olanzapine medication to evaluate its effectiveness. We observed that the mean YMRS score was significantly reduced after 4 weeks of medication with olanzapine. Furthermore, after 8 weeks of treatment, the mean YMRS score was significantly lower than the mean YMRS score after 4 weeks of medication (Figure 1). We also compared the mean relative quantification (RQ) levels before and after olanzapine treatment in order to investigate the effects of olanzapine on *PDLIM5* mRNA expression in PBLs. We showed that there was no significant difference between *PDLIM5* mRNA expression before and after 4 and 8 weeks of medication (Figure 2). However, we observed that *PDLIM5* mRNA expression was significantly lower in BPD patients than in control subjects (Figure 2). Because it is well known that genetic variation can cause considerable differences in the response to medications among different ethnicities [29], we stratified the sample by ethnicity. The results indicated that there was a significant association between *PDLIM5* mRNA expression and the occurrence of BPD in the Indian patients (*p*=0.028), with a trend towards a positive association in the Chinese BPD patients (*p*=0.064). No association was found between *PDLIM5* mRNA expression and the occurrence of BPD in the Malay patients (*p*=0.289). No further ethnic-based analysis was performed following stratification into ethnic groups because of the small sample size. It should be noted that, from the YMRS scores, our clinician found that all 16 multi-ethnic patients were responsive to olanzapine medication, hence indicating that in this small population of multi-ethnic subjects, there was no suggestion of ethnic variation in response to medication.

No significant correlation was observed between *PDLIM5* mRNA expression and YMRS scores before and after adjustment for age, gender, and ethnicity or before and after 4 and 8 weeks of olanzapine treatment (Table 3). No significant association was observed between *PDLIM5* mRNA expression and duration of illness (*F*=1.156, *p* =0.37), previous non-compliance (*F*=0.233, *p*=0.638), or previous substance abuse (*F*=0.644, *p*=0.437), but a positive trend was observed towards a significant association with a family history of mental illness (*F*=3.427, *p* =0.087), as indicated by the finding that 45% of the patients had a history of mental disease.

**Table 3:** **Correlation between YMRS scoreand RQ level**

**YMRS score vs. RQ**	**Non-adjusted**		**Adjusted for age, gender,and ethnicity**	
	**Pearson correlation**	***p*value (2-tailed)**	**Pearson correlation**	***p*value (2-tailed)**
Before medication	−0.252	0.365	−0.236	0.460
At 4 weeks	−0.180	0.539	−0.216	0.523
At 8 weeks	−0.352	0.262	−0.210	0.587

*RQ* relative quantification, *YMRS* Young Mania Rating Scale.

## Discussion

The mechanism of antipsychotic drug action at the receptor level in reducing the symptoms of mania in BPD is well explained as being due to the blockade of the D2 receptor; the antidepressant effect of the antipsychotics, on the other hand, relates to the action of the 5-HT receptors [30]. However, the effect of antipsychotics on events downstream of the receptor is unclear. Many studies relate mood disorder to abnormalities of neuroplasticity in the central nervous system. Changes in the cellular signalling cascade are an example of a neuroplastic event. The cellular signalling cascade plays an important role in the regulation of neuropeptide and neurotransmitter systems and is also a target of psychotropic drugs and hormones that are implicated in the pathophysiology of BPD. Abnormality in the signalling cascade could explain various neurovegetative symptoms and medical comorbidity associated with BPD [31]. Olanzapine has been shown to activate certain signalling cascade such as the JAK/STAT, Akt/PKB, p38, and ERK1/2 pathways [32]. From these findings, it was suggested that activation of the signalling cascade by olanzapine could alter the expression of genes involved in the signalling cascade, leading to a positive therapeutic effect [32].

We hypothesized that olanzapine administration causes changes in the expression of *PDLIM5* mRNA, which encodes a protein that functions in the regulation of PKC activity, leading to improvement of manic symptoms in BPD. We observed a significant reduction in YMRS scores over 8 weeks of medication, indicating the effectiveness of olanzapine medication in this BPD group. Previous studies have also confirmed the effectiveness of olanzapine in the treatment of BPD [23,24,33]. However, we observed no significant changes in *PDLIM5* mRNA expression over 8 weeks of treatment with olanzapine, nor did we find any correlation between improvement in manic symptoms of BPD and *PDLIM5* mRNA expression. Interestingly, in a study of schizophrenia patients, Numata *et al.*[12] showed that the expression of *PDLIM5* was significantly higher in the leukocytes of treatment-naïve patients than it was in control subjects. However, when these investigators compared *PDLIM5* expression between controls and schizophrenia patients who were stable after receiving antipsychotic medication for at least 3 months, they observed no significant difference. They reported that this finding may be a consequence of the pharmacological effects of antipsychotics or of clinical improvement and suggested that the expression of *PDLIM5* mRNA may not be a trait-oriented change, but a state-related one that may respond to environmental factors such as antipsychotic medication [12]. It is notable that the study was cross-sectional and that no specific antipsychotic medication was mentioned. In contrast, our study did not show any correlation between *PDLIM5* expression and olanzapine treatment. These contrasting findings in the association between antipsychotics and *PDLIM5* expression may relate to differences in the disease condition, that is, BPD and schizophrenia, or to antipsychotic medication other than olanzapine. Our results indicate a lack of interaction between PDLIM5 and olanzapine medication in improving the manic symptoms of BPD.

Gene expression in peripheral blood is assumed to reflect the expression of the gene in the prefrontal cortex, making it potentially useful as a surrogate marker for mental disorder [34]. Moreover, components of the immune system found in peripheral blood, such as cytokines, lymphocytes, and interleukins, have also been found in the central nervous system [34]. Thus, studying mRNA expression in peripheral blood has been suggested to reveal disease-specific immune changes and provide a marker of disease progression. In our study, we observed that *PDLIM5* mRNA expression in PBLs was significantly reduced in treatment-naïve BPD patients compared with that in healthy control subjects. Our results support previous findings that showed a significant decrease in *PDLIM5* mRNA expression in the lymphoblastoid cell lines of BPD and schizophrenia patients [14,15], as well as in the PBLs of patients with major depressive disorder [13], compared with that in control subjects. Our results also support previous studies that suggested that the difference in expression level of the *PDLIM5* gene in PBLs between patients and control subjects could be used as a potential biological marker for mental disorder [12,13]. Further functional studies with larger sample sizes are needed to confirm the effectiveness of PDLIM5 in such a role.

One important factor that could affect gene expression is genetic polymorphism. To examine the effect of SNPs on the expression level of *PDLIM5* mRNA in untreated BPD, we genotyped four SNPs of *PDLIM5* that have previously been found to be significantly associated with BPD [6,7]. We observed no significant correlation between these four SNPs, located in the upstream region of the *PDLIM5* gene, with *PDLIM5* mRNA expression in peripheral leukocytes. From these preliminary data, we conclude that none of these SNPs significantly affected the expression of *PDLIM5* mRNA in PBLs. However, we cannot exclude the possibility of a type II error, as our sample size in this association study was relatively small.

## Conclusion

In summary, we showed that there was no correlation between *PDLIM5* mRNA expression and clinical improvement in BPD patients during 8 weeks of olanzapine treatment. We also found that *PDLIM5* mRNA expression in PBLs was significantly lower in treatment-naïve patients than in control subjects, but found no association between each of the four SNPs of *PDLIM5* and mRNA expression level. Our results suggest that further studies are warranted of *PDLIM5* mRNA expression in PBLs as a potential biological marker for BPD.

## Competing interests

The authors have seen and agreed with the contents of the manuscript and declare that they have no competing interests.

## Authors’ contributions

MAZ carried out the study design, performed gene expression and statistical analysis, and drafted the manuscript. SNJ was involved in sample collection and evaluation of patients and controls. GPR and ZM helped to improve the study design and to critically review and draft the manuscript. SK and ZZ provided clinical advice regarding patients and were involved in applying for ethics permissions and grant funding, negotiating with drug companies, and managing the collection of case samples in hospital. All authors read and approved the final manuscript.

## Pre-publication history

The pre-publication history for this paper can be accessed here:

http://www.biomedcentral.com/1471-2350/13/91/prepub
